# GENETIC ARCHITECTURE OF SUBJECTIVE HEALTH: RELATIONSHIP WITH PHYSICAL HEALTH, COGNITION, AND DEPRESSION

**DOI:** 10.1093/geroni/igac059.1751

**Published:** 2022-12-20

**Authors:** Deborah Finkel, Margaret Gatz

**Affiliations:** Indiana University Southeast, New Albany, Indiana, United States; University of Southern California, Los Angeles, California, United States

## Abstract

The fact that self-rated health (SH) predicts mortality and a variety of other health outcomes independent of objective health measures generates questions about mechanisms and etiologies. SH can be considered an indicator of physical health, per se, resulting from active cognitive processing of explicit information about one’s own health and intuitive knowledge of symptoms and physical sensations. The extent to which SH taps shared cultural ideas about health should be reflected in estimates of the shared environmental component of variance (C). SH has also been associated with emotional health measures, such as neuroticism and depression. Previous analyses have been limited by sex (only women), sample size, age (range = 63-76), and failure to include cognitive function. The current analysis used data from 8291 adults ranging in age from 22 to 102 from the international Interplay of Genes and Environment Across Multiple Studies (IGEMS) consortium to investigate the genetic architecture of SH. Genetic influences on self-rated health (SRH) were investigated in the context of CIRS (Cumulative Illness Rating Scale), MMSE (Mini-Mental Status Exam), and depression (CES-D or CAMDEX). Independent pathways modeling indicated that all genetic variance for SRH was shared with CIRS, MMSE, and depression. Comparison of groups older and younger than 74 indicated age differences in genetic architecture of SRH. Evidence suggests that the discordance between objective and subjective health increases in late adulthood, possibly as a result of greater emphasis on psychological rather than physical components of subjective health assessments by older adults.

